# Temperature-Change-Based Thermal Tomography

**DOI:** 10.1155/2009/464235

**Published:** 2009-07-22

**Authors:** Yong Xu, Xiangyu Wei, Ge Wang

**Affiliations:** ^1^Department of Electrical and Computer Engineering, Virginia Polytechnic Institute and State University, Blacksburg, VA 24061, USA; ^2^School of Biomedical Engineering and Sciences, Virginia Polytechnic Institute and State University, Blacksburg, VA 24061, USA

## Abstract

Thermal properties of biological tissues play a critical role in the study of tumor angiogenesis and the design and monitoring of thermal therapies. To map thermal parameters noninvasively, we propose temperature-change-based thermal tomography (TTT) that relies on relative temperature mapping using magnetic resonance imaging (MRI). Our approach is unique in two aspects: (1) the steady-state body temperature in thermal equilibrium is not restricted to be spatially invariant, and (2) absolute temperature mapping is not required. These two features are physiologically realistic and technically convenient. Our numerical simulation indicates that a (9 mm)^3^ tumor inside a breast phantom can be reliably depicted, assuming moderate temperature mapping accuracy of 0.5°C.

Traditional thermal tomography is based on using an infrared camera to measure the surface temperature of a tissue and then solving the inverse heat transfer problem to reconstruct the interior tissue thermal parameters [1]. Since 2002, a few papers [2–4] have discussed the possibility of using magnetic resonance imaging (MRI) to obtain the temperature distribution *within* the tissue and reconstruct the temperature coefficients therein. Current approaches in literature, however, have a few significant drawbacks. First of all, the theoretical model in previous work [2–4] is based on the original Pennes' bioheat transfer equation [5], which requires absolute-temperature measurements. Yet most of the MRI-based temperature-mapping techniques, such as those based on proton resonance frequency (PRF), can only provide relative-temperature-change [6]. Second, the reconstruction algorithms in previous work [3, 4] are mathematically complicated. Last but not least, at thermal equilibrium, the in vivo temperature distribution in the region of interest (ROI) is often nonuniform. For example, the presence of a diseased tissue such as a tumor often leads to increased metabolic activity and an abnormal temperature distribution. In particular, clinical studies have suggested that the local skin temperature over a breast tumor can increase significantly (by 1–3°C) over the normal breast skin temperature [7]. This effect is often ignored in current analyses, which assume that the steady-state temperature within the ROI is uniform. To address these deficiencies, we formulate a temperature-change-based thermal tomography (TTT) method and demonstrate that TTT can potentially lead to new and noninvasive medical modalities for breast cancer imaging and other important biomedical applications.

Assuming absolute temperature within a biological tissue is described by the Pennes' bioheat transfer equation, we have

(1) $$\;\rho C_{T}\frac{\partial T}{\partial t}=\nabla \cdot \left(\kappa \nabla T\right)-\omega_{B}C_{B}\left(T-T_{A}\right)+Q_{\text{int}}+Q_{\text{ext}},$$

where *T* is the absolute temperature, *κ* is tissue thermal conductivity, *T*_*A*_ is the normal body temperature, *ω*_*B*_ is the blood perfusion rate, *c*_*T*_ and *c*_*B*_ are the specific heat of tissue and blood, respectively, and *Q*_int_ and *Q*_ext_ are the internal and external heat sources, respectively. In the absence of external heat sources, the absolute temperature distribution *T*_*S*_ at thermal equilibrium satisfies ∇ · (*κ*∇*T*_*S*_) − *ω*_*B*_ *C*_*B*_(*T*_*S*_ − *T*_*A*_) + *Q*_int_ = 0. We note that the steady-state temperature *T*_*S*_ may not be uniform within the ROI. However, the impacts due to nonuniform *T*_*S*_ can be eliminated through relative temperature measurements. In relative temperature measurements, we first obtain a baseline MRI image at thermal equilibrium, which can be accomplished in vivo and noninvasively. We then thermally excite the sample and use MRI to record the thermal processes within the biological tissue. From temperature sensitive parameters such as proton resonance frequency [6], we can extract the temperature change Δ*T* by comparing the MRI images taken at a later time and the baseline MRI image. Since the absolute temperature *T*_*S*_ + Δ*T* satisfies (1), we can recast the Pennes' bioheat transfer equation for the relative temperature Δ*T* as

(2) $$\frac{\partial \Delta T}{\partial t}=\nabla \cdot \left[\alpha_{1}\nabla \left(\Delta T\right)\right]-\alpha_{2}\Delta T+\frac{1}{\rho C_{T}}Q_{\text{ext}},$$

where *α*_1_ is thermal diffusivity and equals to *κ*/*ρC*_*T*_, *α*_2_ is blood perfusion parameter and is given by *C*_*B*_ *ω*_*B*_/*ρC*_*T*_, and *Q*_ext_ represents heat generated by external sources (e.g., focused ultrasound). Now we continuously perform relative temperature measurements from time *t*_0_ and to time *t*_1_. Since Δ*T* satisfies (2), after integrating both sides of (2) from *t*_0_ to *t*_1_ in the absence of external sources, we obtain

(3) $$\nabla \cdot \left(\alpha_{1}\nabla \langle \Delta T\rangle \right)-\alpha_{2}\langle \Delta T\rangle =\frac{T\left(t_{1}\right)-T\left(t_{0}\right)}{\Delta t},$$

where Δ*t* = *t*_1_ − *t*_0_, and 〈Δ*T*〉 is the time average of the relative temperature, that is, 〈Δ*T*(*x*, *y*, *z*)〉 ≡ ∫_*t*_0__^*t*_1_^Δ*T*(*x*, *y*, *z*, *t*)*dt*/Δ*t*. If we discretize the spatial domain of the biological structure into *N* grid points and apply (3) at each point, we obtain *N* linear equations with 2*N* unknown variables (one *α*_1_ and one *α*_2_ at each grid point). Performing an additional set of MRI temperature measurements with a different initial temperature, we have 2*N* linear equations and can uniquely determine the values of *α*_1_ and *α*_2_ over the entire numerical grid. Such a direct approach for TTT is significantly simpler than other thermal reconstruction methods in literature [2–4].

Having established the general framework of TTT, we consider an important practical application: the imaging and detection of breast cancer. It has been shown that blood flow in tumors and in normal tissues can differ dramatically [1, 2]. For example, the blood perfusion rate **ω*_B_* of normal breast tissue is 0.7 kg · m^−3^ · s^−1^ [8], whereas *ω*_*B*_ for breast adenocarcinoma is 5 ~ 6.7 kg · m^−3^ · s^−1^ [2]. Assuming a tissue density *ρ* of 1 × 10^3^ kg · m^−3^, a tissue specific heat of *c*_*T*_ = 3550 J · kg^−1^ · °C^−1^ [1, 9], and a blood specific heat of *c*_*B*_ = 3600 J · kg^−1^ · °C^−1^ [1], the blood perfusion parameter *α*_2_ for normal breast is 7.1 × 10^−4^ s^−1^, whereas for breast tumor, *α*_2_ ranges from 5.1 × 10^−3^ s^−1^ to 6.8 × 10^−3^ s^−1^. Notice that the value of *α*_2_ in cancerous tissue increases by almost one order of magnitude. Thermal conductivity *κ*, however, remains similar in cancerous and normal tissues. For example, the values of *κ* for tumor, lung, and skeletal muscle are 0.642 [9], 0.30 ~ 0.55 [1], and 0.45 ~ 0.55 W · m^−1^ · °C^−1^ [1], respectively. In this letter, we assume that *κ* is 0.47 W · m^−1^ · °C^−1^ for both the tumor and the normal tissue, which gives a thermal diffusivity *α*_1_ of 0.13 mm^2^ · s^−1^. With these considerations, a breast phantom is established in Figure 1: the phantom is a hemisphere with a radius of 75 mm and contains a (9 mm)^3^ tumor buried within. The thermal diffusivity *α*_1_ is 0.13 mm^2^ · s^−1^ throughout the phantom (including the tumor). The blood perfusion parameter *α*_2_ is 5.6 × 10^−3^ s^−1^ within the tumor and 7.1 × 10^−4^ s^−1^ outside of it.

Now we use a finite-difference (FD) method [10] to simulate temperature evolution within the breast phantom. (Later on, the simulated temperature field is used for thermal reconstruction tests). The simulation domain, which is illustrated in Figure 1, has a dimension of 150 × 150 × 84 mm^3^. It is evenly divided into an array of FD cells with a cell size of Δ*x* = Δ*y* = Δ*z* = 3 mm. The number of cells along the *x*, *y*, and *z* direction is, respectively, 50, 50, and 28. Each FD cell is labeled by three indices as (*i*, *j*, *k*), with cell center located at the position of (*i*Δ*x*, *j*Δ*y*, *k*Δ*z*). The center of the breast phantom is at cell (25, 25, 28). The tumor region is represented by 9 FD cells whose indices are in the range of 27 ≤ *i* ≤ 29, 19 ≤ *j* ≤ 21, and 18 ≤ *k* ≤ 20. The FD simulation is carried out by discretizing (2) and evolving the temperature field forward with a time step of Δ*t* = 1 second. For boundary condition, we set the relative temperature shift at the breast boundary to be zero. This assumption is reasonable, since the temperature at the breast surface can be kept at a constant value and deep within the breast (at the top *z* surface) the temperature shift should be minimal. For the initial temperature at time *t*_0_, we assume that it has a Gaussian profile Δ*T*(*x*, *y*, *z*) = Δ*T*_0_ exp{−[(*x* − *x*_0_)^2^ + (*y* − *y*_0_)^2^ + (*z* − *z*_0_)^2^]/*δ* ^2^} with the following parameters: Δ*T*_0_ = 5°C, *x*_0_ = 25Δ*x*, *y*_0_ = 24Δ*y*, *z*_0_ = 17Δ*z*, and *δ* = 9Δ*x*. In Figure 2, we show a few snapshots of the relative temperature field obtained through FD simulations. The results in Figure 2(a) are plotted along a straight line that passes through the tumor center and is along the *x* direction, that is, the dashed line in Figure 1. Since MRI has a finite accuracy in temperature measurement, to make our analysis more realistic, we add Gaussian noises with a standard deviation of 0.5°C to the simulation results. (A measurement accuracy of 0.5°C with a spatial resolution of ~1 mm is realistic given current MRI technology [2]). Figure 2(b) gives the temperature field at *t* = 200 s, one from FD simulation and the other with noise added. Figure 2(c) shows the temporal evolution of the noise-added temperature field at cell (28, 20, 19), the center of the tumor.

We now treat the noise-added simulation results as MRI measurements data and use them to test our thermal reconstruction algorithm. In this letter, we assume that *α*_1_ is known to be 0.13 mm^2^ · s^−1^ throughout the breast phantom, which means that we only need one set of temperature measurements to reconstruct the blood perfusion parameter *α*_2_. This assumption is reasonable, since the value of *α*_1_ does not vary significantly from tumor to normal tissues. Furthermore, the importance of the thermal diffusion term can be greatly reduced by choosing a relatively flat temperature distribution, which is the case for the initial Gaussian field used in Figure 2. To further illustrate this point, consider an extreme case: for a uniform temperature distribution, the thermal diffusion term in (2) becomes zero, and the value of *α*_1_ has no impact on the temperature decay rate.

In the absence of noise, the spatial distribution of *α*_2_ can be directly obtained through (3). However, if the input temperature-change data Δ*T* contain significant noises, a straightforward application (3) is not advisable. In fact, a finite-difference evaluation of ∇ · (*α*_1_∇〈Δ*T*〉) can significantly amplify the already nonnegligible measurement noises. Fortunately, we can use numerical fitting to achieve significant noise reduction. As an example, we notice that with a uniform distribution of *α*_1_, ∇ · (*α*_1_∇〈Δ*T*〉) becomes *α*_1_∇^2^〈Δ*T*〉, which can be evaluated by polynomial fitting. More specifically, to evaluate ∂^2^〈Δ*T*〉/∂*x* ^2^ at cell (*i*_0_, *j*_0_, *k*_0_), we use the polynomial 〈Δ*T*〉 = *a*_2_(*i* − *i*_0_)^2^Δ*x* ^2^ + *a*_1_(*i* − *i*_0_)Δ*x* + *a*_0_ to fit the temperature data at cells (*i*, *j*_0_, *k*_0_), with *i* in the range of *i*_0_ − 2 ≤ *i* ≤ *i*_0_ + 2. After fitting, the value of ∂^2^〈Δ*T*〉/∂*x* ^2^ is simply given by the fitting coefficient *a*_2_. ∂^2^〈Δ*T*〉/∂*y* ^2^ and ∂^2^〈Δ*T*〉/∂*z* ^2^ can be similarly evaluated. Next, we consider the evaluation of *T*(*t*_1_) − *T*(*t*_0_) in (3). To eliminate measurement noises at any specific time, we use an exponential function to fit the temporal evolution of the temperature field. In Figure 2(c), we show the fitting result (represented by a dashed line) at cell (28, 20, 19). (The original noise-added temperature data are represented as dots.) As can be seen from Figure 2(c), the exponential fitting provides a reasonable approximation to the noise-added temperature data. We can then use the fitted curve to calculate *T*(*t*_1_) − *T*(*t*_0_). After applying the aforementioned fitting schemes to (3), we can reconstruct the spatial distribution of the blood perfusion parameter *α*_2_ from the noise-added FD simulation data. The reconstruction results are shown in Figure 3 as diamonds. (The data are plotted along the dashed line in Figure 1, which is the same as the line used in Figure 2). We repeat the same reconstruction procedure 30 times, each time using the same FD simulation data with randomly generated Gaussian noises. From the statistical fluctuations of the reconstructed *α*_2_, we can obtain the standard deviations for *α*_2_, which are given in Figure 3 as error-bars. We also apply the same reconstruction procedure to the FD simulation data *without* any noise. The results are shown in Figure 3 as dots connected by solid lines. The input *α*_2_ values (used in forward FD simulations) are also shown in Figure 3 as a dashed line. From Figure 3, we find that despite the 0.5°C random noises, we can successfully reconstruct the spatial distribution of the blood perfusion parameter *α*_2_. We also observe that the numerical fitting introduces approximately 20% systemic errors to the thermal reconstruction. However, it is possible to reduce the systemic error by developing a more accurate noise reduction algorithm.

We emphasize that the advantages of TTT are derived primarily from its relative-temperature basis. By formulating a differential equation based on temperature changes (i.e., (2)), we remove any potential impact due to an abnormal temperature profile in the ROI at thermal equilibrium. Furthermore, the requirement of relative temperature measurements makes TTT much more suitable for clinical applications.

The algorithm presented in this paper serves as a proof-of-concept validation and can be improved in future studies. For example, the method of five-point polynomial fitting can be replaced by other spatial filtering techniques that can effectively reduce the measurement noises. The exponential temporal fitting can also be replaced by more sophisticated filtering techniques such as the Wiener filter. The assumption of an initial Gaussian temperature distribution is also not necessary: We have verified the reconstruction algorithm (i.e., (3)) using different initial temperature distribution and other means of thermal excitations.

Compared with other methods of thermal tomography in current literature, the method presented in this paper is significantly simpler. For example, the method presented in [3] relies on the framework of Bayesian statistics and statistical inverse problems. Yet in current work, we can achieve similar resolution using a much simpler numerical approach. We also point out that within a temperature range of less than 4°C (refer to Figure 2), tissue thermal parameters should remain approximately the same [9]. Consequently, the effects of nonlinear heat transfer should not significantly change the main conclusions of this work. As presented in current paper, TTT may require an image acquisition time of a few minutes, during which the effects of motion artifacts may arise. Such artifacts, however, can be addressed using methods reviewed in [6].

In conclusion, we have developed a temperature-change-based thermal tomography and established a simple and straightforward thermal reconstruction algorithm. Using realistic thermal parameters and assuming a temperature measurement accuracy of 0.5°C, we have successfully reconstructed the spatial distribution of a (9 mm)^3^ tumor embedded in a human breast phantom. We believe that TTT can lead to new and noninvasive medical modalities for cancer detection and imaging.

## Figures and Tables

**Figure 1:**
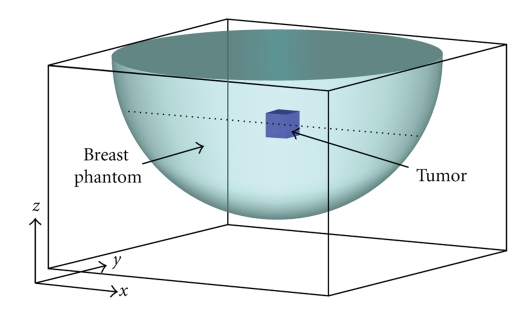
Schematic of the breast phantom.

**Figure 2:**
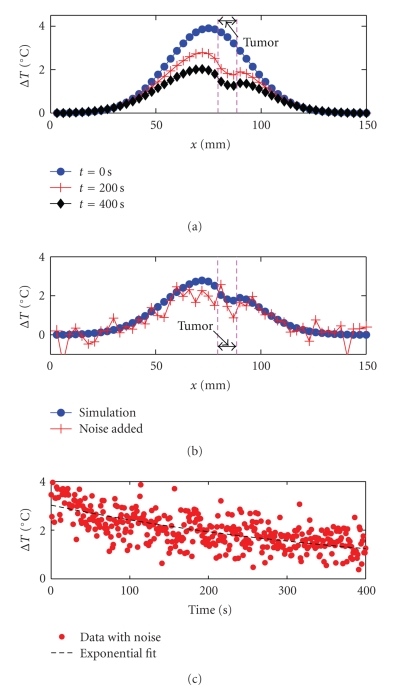
(a) Relative temperature field plotted along the dashed line in Figure 1, at time *t* = 0, *t* = 200 seconds, *t* = 400 seconds, respectively. (b) Relative temperature field at *t* = 200 seconds. The dots and the crosses represent FD simulation data and noise-added data, respectively. (c) Time evolution of the noise-added temperature field at FD cell (28, 20, 19). The dashed line is an exponential fit.

**Figure 3:**
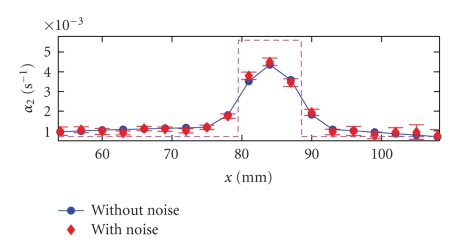
Reconstructed thermal coefficients *α*_2_ along the dashed line in Figure 1.
